# Comparing Multivisceral Resection with Tumor-only Resection of Liposarcoma Using the Win Ratio

**DOI:** 10.1245/s10434-024-14985-8

**Published:** 2024-02-12

**Authors:** Leva Gorji, Melica Nikahd, Amblessed Onuma, Diamantis Tsilimigras, J. Madison Hyer, Samantha Ruff, Farhan Z. Ilyas, Carlo Contreras, Valerie P. Grignol, Alex Kim, Raphael Pollock, Timothy M. Pawlik, Joal D. Beane

**Affiliations:** 1 Department of Surgery, Kettering Health Dayton, Dayton, OH USA; 2 Department of Biomedical Science-Biomedical informatics Columbus, Columbus, OH USA; 3 Department of Surgery, The Ohio State University Wexner Medical Center Columbus, Columbus, OH USA; 4 Division of Surgical Oncology, Department of Surgery, The Ohio State University Wexner Medical Center and James Cancer Hospital, Columbus, OH USA; 5 College of Medicine, The Ohio State University Columbus, Columbus, OH USA

**Keywords:** Multivisceral resection, Tumor-only resection, Retroperitoneal soft tissue sarcoma

## Abstract

**Background:**

Multivisceral resection of retroperitoneal liposarcoma (LPS) is associated with increased morbidity and may not confer a survival benefit compared with tumor-only (TO) resection. We compared both approaches using a novel statistical method called the “win ratio” (WR).

**Methods:**

Patients who underwent resection of LPS from 2004 to 2015 were identified from the National Cancer Database. Multivisceral resection was defined as removal of the primary site in addition to other organs. The WR was calculated based on a hierarchy of postoperative outcomes: 30-day and 90-day mortality, long-term survival, and severe complication.

**Results:**

Among 958 patients (multivisceral 634, TO 324) who underwent resection, the median age was 63 years (interquartile range [IQR] 54–71) with a median follow-up of 51 months (IQR 30–86). There was no difference in the WR among patients who underwent TO versus multivisceral resection in the matched cohort (WR 0.82, 95% confidence interval [CI] 0.61–1.10). In patients aged 72–90 years, those who underwent multivisceral resection had 36% lower odds of winning compared with patients undergoing TO resection (WR 0.64, 95% CI 0.40–0.98). A subgroup analysis of patients classified as not having adjacent tumor involvement at the time of surgery revealed that those patients who underwent multivisceral resection had 33% lower odds of winning compared to TO resection (WR 0.67, 95% CI 0.45–0.99).

**Conclusions:**

Based on win-ratio assessments of a hierarchical composite endpoint, multivisceral resection in patients without adjacent tumor involvement may not confer improved outcomes. This method supports the rationale for less invasive resection of LPS in select patients, especially older patients.

Retroperitoneal soft tissue sarcomas (RPS) are rare tumors comprising only 0.2% of adult malignancies.^1,2^ These neoplasms account for approximately 10–20% of a larger group of heterogenous malignancies known as soft tissue sarcomas, which often are plagued by local morbidity rather than metastasis.^2,3^ Liposarcomas (LPS) are the most common variant and account for more than 50% of all RPS.^4^ Because of the ample potential space of the retroperitoneum, LPS often remains clinically silent until reaching large dimensions; initial tumor size ranges from 5 to 45 cm on diagnosis.^5–7^ At least five subtypes of liposarcomas exist, including well-differentiated, de-differentiated, myxoid, round cell, and pleomorphic with the well-differentiated and de-differentiated subtypes being the most common.^4^ Notably, well-differentiated LPS has the most favorable prognosis of the histologic subtypes.^8^

Surgical resection with grossly negative margins is a good prognostic indicator and the only potentially curative approach currently available.^9–12^ Management remains a challenge as nearly 50% of primary retroperitoneal liposarcomas recur within 5 years.^9,10^ In select patient populations, systemic therapy and radiation may be used as adjuncts to surgery.^11–13^

Two operative strategies have evolved over the past decade and include multivisceral resection and tumor-only (TO) resection. Multivisceral resection requires excision of the tumor in addition to adjacent organs abutting the tumor, even in the absence of direct invasion. For example, the ipsilateral kidney, segment of the colon, and left upper quadrant organs for patients with large left-sided RPS. Aggressive surgical intervention, including en bloc resection of uninvolved, adjacent organs, has been employed to optimize surgical margins, prevent local recurrences, and extend overall survival (OS).^14,15^ However, in some studies, the demanding pursuit of multivisceral, en bloc resection resulted in increased morbidity without significant improvements in OS.^14,16^ In contrast, a TO resection for patients with RPS is limited to excision of the RPS with negative margins. Proponents of this approach tout a reduction in surgical morbidity and comparable oncologic outcomes.^14^ In this study, we compare multivisceral resection versus TO resection of LPS using the win ratio (WR), a novel approach that analyzes composite endpoints of two competing approaches. This method allows for flexibility in prioritizing endpoints of analysis, which allows for the evaluation of postoperative outcomes in a hierarchical order to define an overall benefit.^17^

## Methods

Patients who underwent resection of RPS from 2004 to 2015 were identified from the National Cancer Database (NCDB) using the International Classification of Diseases (ICD-O-3) third edition. The following histology subtypes were included in the search: dedifferentiated liposarcoma (DDLPS), well-differentiated liposarcoma (WDLPS), and liposarcoma not-otherwise specified (LPS NOS). Among patients with this diagnosis, those who underwent an operation were identified using NCDB surgical site code C48.0, which identifies malignant neoplasm of the retroperitoneum. As previously described by Villano et al., only surgical codes that represented curative intent surgery (code 40 = total surgical removal of primary site, code 60 = radical surgery) were included in the final analysis.^14^ Patients with R2 resection and stage IV malignancies were excluded from the final analysis to allow for the selection of patients undergoing curative-intent operation.

Multivisceral resection was defined as partial or total removal of the primary site with a resection in continuity with other organs. Adjacent tumor involvement was determined based on the “Collaborative Stage-Extension” codes in NCDB; codes 60, 80, 600, and 800 were used to identify LPS tumors with adjacent tumor involvement. Of note, this variable has been externally validated by Villano et al. in a surgically treated cohort of patients with colorectal cancer within the NCDB and published.^14^

For the win ratio (WR), all possible combinations of patients with multivisceral resection and TO resection were paired and kept only if the pair of patients had the same clinical presentation (i.e., the same values for adjacent tumor involvement, grade, stage, age group (based on quartiles), and Charlson-Deyo Comorbidity Index (± 1 unit)). After matching, the win ratio (WR) was used to assess the effect of resection status on the odds of achieving a “win” after resection of LPS.^18,19^ This was performed by first ranking postoperative outcomes in the following order: 30-day mortality > 90-day mortality > long-term survival (overall survival) > severe complications (defined by readmission within 30 days of surgery). Beginning with 30-day mortality, a WR was calculated at each tier as the (number of “wins”)/(number of “losses”), and 95% confidence intervals [CI] were calculated by using bootstrapping methodology. For 30-day mortality, 90-day mortality, and severe complications, a win was defined as the absence of the postoperative outcome for multivisceral resection group and the presence of the outcome in the TO resection group, whereas a loss was defined as the presence of the outcome in the multivisceral resection group and the absence of the outcome in the TO resection group. When both pairs experienced 30- or 90-day mortality, no further comparisons were made in the lower tiers. For long-term survival, if the deceased multivisceral resection pair had longer survival than the deceased TO resection pair, then that was classified as a win, and the converse was defined as a loss. If survival was equal or neither patient died in the pair, then further comparisons were made in the lower tiers. Unstratified WRs and 95% CIs were presented, as well as stratified WRs for sex, race, age group, tumor grade, tumor stage, histology subtype, and adjacent tumor involvement. A power analysis was conducted by using the unmatched pair method. Significance was assessed at the 0.05 level, and all analyses were performed by using SAS 9.4.

Descriptive statistics included median and interquartile range (IQR) for continuous variables and frequencies and percentages for categorical variables. A bivariate analysis between each covariate and extent of resection was performed by using chi-square test for categorical variables, or Fisher’s exact test when expected counts were ≤ 5; bivariate analyses for continuous covariates were assessed using Mann-Whitney *U* test. For each tier used to calculate the win ratio, logistic and Cox regression also were provided to describe differences between multivisceral and tumor-only resection.

## Results

Among 967 patients who underwent resection with curative intent, the median age was 63 years (interquartile range [IQR] 54–71) with a median follow-up of 51 months (IQR 30–86) (Table 1). Α total of 639 (66%) patients underwent multivisceral resection for LPS, whereas 328 (34%) patients underwent TO resection. Nine patients were excluded at the time of analysis because of missing values for 90-day mortality. The final cohort consisted of 634 patients in the multivisceral resection cohort and 324 patients in the TO resection cohort. Most of the cohort was white (*n* = 857, 88.6%), and approximately half was comprised of male patients (*n* = 533, 55.1%). Approximately one-third of the patients (*n* = 371, 38.4%) had their operation at a high-volume facility, defined as those with ten or more LPS resections per year placing them in the 95th percentile of surgeries per year for this cohort (Table 1). Furthermore, a total of 667 patients (70%) underwent surgical intervention at an academic center. Academic centers were defined as academic/research program facilities, and Community Cancer Programs (CCP), Comprehensive Community Cancer Programs (CCCP), and Integrated Cancer Programs (INCP) were categorized as nonacademic centers.^20,21^

**Table 1:** Sample demographic and clinical characteristics by extent of resection (*N* = 967)

	Tumor-only (*n* = 328)	Multivisceral (*n* = 639)	Total (*N* = 967)	*p*
	Demographics			
Male, *n* (%)	186 (56.7%)	347 (54.3%)	533 (55.1%)	0.48
Age at diagnosis, median (IQR)	64 (56, 72)	63 (54, 71)	63 (54, 71)	0.19
Age group, *n* (%)				
40–54 years	76 (23.2%)	168 (26.3%)	244 (25.2%)	0.38
55–63 years	79 (24.1%)	161 (25.2%)	240 (24.8%)	
64–71 years	82 (25.0%)	164 (25.7%)	246 (25.4%)	
72–90 years	91 (27.7%)	146 (22.8%)	237 (24.5%)	
Race, *n* (%)				
White	290 (88.4%)	567 (88.7%)	857 (88.6%)	0.21
Black	22 (6.7%)	29 (4.5%)	51 (5.3%)	
Other	16 (4.9%)	43 (6.7%)	59 (6.1%)	
	Clinical			
Charlson-Deyo comorbidity index, *n* (%)				
0	236 (72.0%)	511 (80.0%)	747 (77.2%)	0.010
1	76 (23.2%)	98 (15.3%)	174 (18.0%)	
2	16 (4.9%)	30 (4.7%)	46 (4.8%)	
Tumor grade, *n* (%)				
I	176 (53.7%)	260 (40.7%)	436 (45.1%)	0.001
II	27 (8.2%)	54 (8.5%)	81 (8.4%)	
III	67 (20.4%)	184 (28.8%)	251 (26.0%)	
IV	58 (17.7%)	141 (22.1%)	199 (20.6%)	
Tumor stage, *n* (%)				
I	186 (56.7%)	267 (41.8%)	453 (46.8%)	< 0.001
II	29 (8.8%)	65 (10.2%)	94 (9.7%)	
III	113 (34.5%)	307 (48.0%)	420 (43.4%)	
Year of diagnosis, *n* (%)				
2004–2007	96 (29.3%)	168 (26.3%)	264 (27.3%)	0.55
2008–2011	109 (33.2%)	212 (33.2%)	321 (33.2%)	
2012–2015	123 (37.5%)	259 (40.5%)	382 (39.5%)	
Histology subtype, *n* (%)				
DDLPS	105 (32.0%)	302 (47.3%)	407 (42.1%)	< 0.001
WDLPS	125 (38.1%)	198 (31.0%)	323 (33.4%)	
LPS	98 (29.9%)	139 (21.8%)	237 (24.5%)	
CS tumor extension, *n* (%)				
Localized, not otherwise specified	1 (0.3%)	0 (0.0%)	1 (0.1%)	< 0.001
Tumor confined to site of origin	82 (25.0%)	114 (17.8%)	196 (20.3%)	
Localized, not otherwise specified	77 (23.5%)	89 (13.9%)	166 (17.2%)	
Adjacent connective tissue involvement	61 (18.6%)	132 (20.7%)	193 (20.0%)	
Adjacent organs/structures*	97 (29.6%)	274 (42.9%)	371 (38.4%)	
Further contiguous extension^†^	10 (3.0%)	30 (4.7%)	40 (4.1%)	
Adjacent Tumor Involvement, *n* (%)	107 (32.6%)	304 (47.6%)	411 (42.5%)	< 0.001
Adjuvant therapy, *n* (%)	12 (3.7%)	24 (3.8%)	36 (3.7%)	0.94
High-volume facility, *n* (%)	95 (29.0%)	276 (43.2%)	371 (38.4%)	< 0.001
Facility Type, *n* (%)				
Community cancer program	15 (4.6%)	5 (0.8%)	20 (2.1%)	< 0.001
Comprehensive community cancer program	90 (27.4%)	99 (15.5%)	189 (19.5%)	
Academic/research program	201 (61.3%)	476 (74.5%)	677 (70.0%)	
Integrated network cancer program	22 (6.7%)	59 (9.2%)	81 (8.4%)	
Academic centers, *n* (%)	201 (61.3%)	476 (74.5%)	677 (70.0%)	< 0.001
Months of follow-up, median (IQR)	55 (32, 94)	51 (29, 83)	52 (30, 86)	0.19
30-day mortality, n (%)	5 (1.5%)	7 (1.1%)	12 (1.2%)	0.57
90-day mortality^‡^, *n* (%)	8 (2.4%)	14 (2.2%)	22 (2.3%)	0.77
Overall mortality, *n* (%)	121 (36.9%)	288 (45.1%)	409 (42.3%)	0.015
Readmission within 30 days of surgical discharge, *n* (%)	25 (7.6%)	50 (7.8%)	75 (7.8%)	0.91
Surgical margins, *n* (%)				
Missing	44 (13.4%)	89 (13.9%)	133 (13.8%)	0.83
Macroscopically negative R0/R1	284 (86.6%)	550 (86.1%)	834 (86.2%)	

*Adjacent organs/structures including bone/cartilage, including the following: adrenal(s), aorta, ascending colon, descending colon, kidney(s), pancreas, vena cava, vertebra

^†^Further contiguous extension, including the following: extension to colon other than ascending/descending

^‡^Total of nine patients with missing data on 90-day mortality

Among the patients who underwent TO resection, 25 (7.6%) patients required readmission within 30 days of surgical discharge, whereas five (1.5%) patients died within 30 days. Among the patients who underwent multivisceral resection, 50 (7.8%) patients required readmission within 30 days of surgical discharge, whereas seven (1.1%) patients died within 30 days. The overall mortality in the TO group was 121 (36.9%) patients compared with 288 (45.1%) patients in the multivisceral resection group. The odds of 30-day mortality were 31% lower in patients with multivisceral resection compared with patients with TO resection (*p* = 0.56); this is actually consistent with findings at tier 1, where the odds of winning were 67% higher for the multivisceral resection group compared to the TO group. The odds of 90-day mortality were 26% lower in patients with multivisceral resection compared with patients with TO resection (*p* = 0.53). Throughout the study period, the risk of death was 10% higher in patients with multivisceral resection compared with patients with TO resection (*p* = 0.40). The odds of severe complications were 20% higher in patients with multivisceral resection compared with patients with TO resection (*p* = 0.49). Additionally, when evaluating trends over time in 4-year increments, the number of patients diagnosed with LPS increased with a higher ratio of patients undergoing multivisceral resection (*p* = 0.55).

**Fig. 1:**
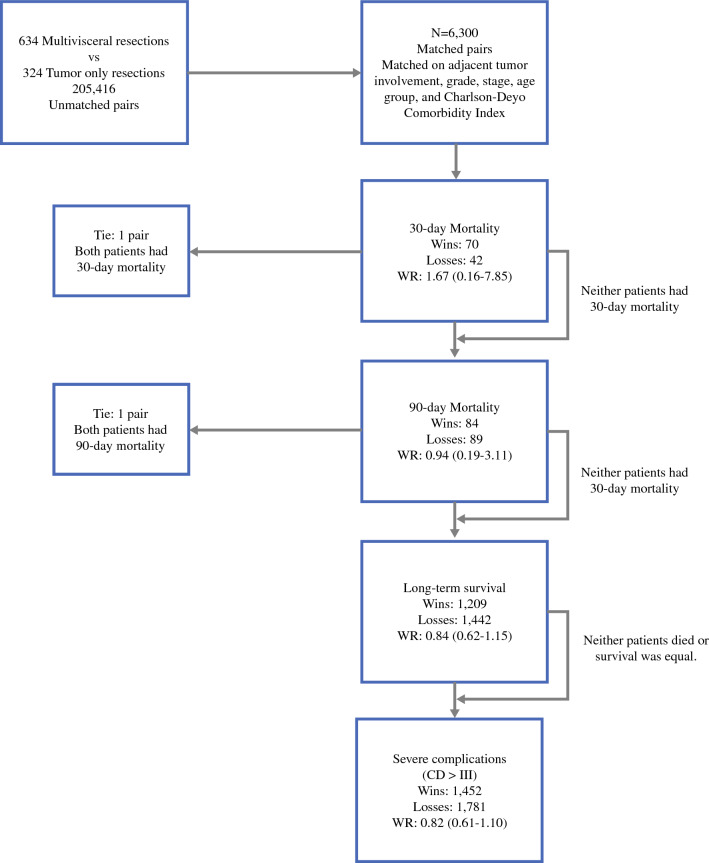
Flowchart demonstrating how the unstratified win ratio was calculated based on the hierarchy of postoperative outcomes

Overall, there was no difference in the WR among patients who underwent multivisceral resection versus TO resection in the matched cohort (WR 0.82, 95% CI 0.61–1.10). Figure 1 presents a decision tree demonstrating how the unstratified WR was calculated given the hierarchy of postoperative outcomes. Table 2 includes the final unstratified WR from Fig. 1 along with the WRs for various subgroups, including stratification for surgical intervention performed at academic centers. In patients aged 72–90 years, those who underwent multivisceral resection had 36% lower odds of winning (WR 0.64, 95% CI 0.40–0.98) compared with patients undergoing TO resection (Table 2). A subgroup analysis of patients classified as not having adjacent tumor involvement at the time of surgery revealed that patients who underwent multivisceral resection had 33% lower odds of winning compared with those who underwent TO resection (WR 0.67, 95% CI 0.45–0.99). Finally, when comparing multivisceral resection to TO resection by each individual outcome used in calculating the WR (30-day mortality, 90-day mortality, severe complication, and long-term survival), there was no significant difference between the two approaches, even while adjusting for adjacent tumor involvement, grade, stage, age, group, and Charlson-Deyo Comorbidity Index (Table 3). Whereas a power analysis of 1000 samples revealed 58.5% power to detect a significant win ratio, there is currently no established way to implement the matched pair method for the win ratio into a power analysis. Because the exact win-ratio method used in this study was not able to be applied to our power analysis, true power may be higher or lower than estimated.

**Table 2:** Stratified and unstratified win ratios (WR) and bootstrapped 95% CIs

	WR (95% CI)
Unstratified	0.82 (0.61–1.10)
Sex	
Male	0.90 (0.59–1.30)
Female	0.85 (0.44–1.37)
Race	
White	0.80 (0.58–1.08)
Black	0.44 (0.00–2.50)
Other	4.24 (0.00–18.00)
Age (year)	
40–54	1.11 (0.46–2.17)
55–63	0.97 (0.48–1.70)
64–71	0.88 (0.46–1.54)
72–90	0.64 (0.40–0.98)
Tumor grade	
I	0.74 (0.46–1.11)
II	0.53 (0.00–2.00)
III	0.90 (0.50–1.40)
IV	1.17 (0.64–1.98)
Tumor stage	
I	0.74 (0.47–1.12)
II	0.50 (0.00–1.79)
III	0.99 (0.68–1.41)
Histology subtype	
DDLPS	0.98 (0.58–1.53)
WDLPS	0.87 (0.45–1.53)
LPS	0.74 (0.34–1.49)
Adjacent tumor involvement	
Yes	1.20 (0.74–1.78)
No	0.67 (0.45–0.99)
High-volume facility	
Yes	0.73 (0.33–1.35)
No	0.93 (0.63–1.30)
Adjuvant therapy	
Yes	1.57 (0.00–8.00)
No	0.82 (0.60–1.09)
Academic centers	
Yes	0.73 (0.49–1.06)
No	0.95 (0.50–1.63)
Year of diagnosis	
2004–2007	1.01 (0.57–1.66)
2008–2011	0.69 (0.36–1.15)
2012–2015	0.76 (0.36–1.31)

*CI* confidence interval

**Table 3:** Effects of multivisceral resection on postoperative outcomes compared to tumor-only resection, adjusted for adjacent tumor involvement, tumor grade, tumor stage, age group, Charlson-Deyo Comorbidity Index, and resection margins

	OR (95% CI)	HR (95%CI)	*p*
30-day mortality	0.69 (0.19–2.45)	–	0.56
90-day mortality	0.74 (0.29–1.90)	–	0.53
Long-term survival	–	1.10 (0.88, 1.37)	0.40
Severe complications	1.20 (0.71–2.02)	–	0.49

*OR* odds ratio, *HR* hazard ratio, *CI* confidence interval

## Discussion

Because of the rarity of RPS and limited studies available pertaining to the malignancy, consensus regarding optimal surgical management is controversial. In this study, we utilized the NCDB and a novel statistical approach called the win ratio to compare 30-day morality, 90-day mortality, overall survival, and readmission within 30 days of surgery in patients who underwent multivisceral versus TO resections. In our analysis of nearly 1000 patients with LPS, we did not find a survival benefit in the use of multivisceral resection. Notably, patients who underwent multivisceral resection between the ages of 72–90 years had lower odds of winning, meaning that this patient population is less likely to have better outcomes compared to a matched cohort who underwent TO resection. The outcomes of this study demonstrate oncological limitations and consequences associated with multivisceral, en bloc resection in patients with retroperitoneal LPS.

Many authors have advocated for aggressive surgical resection with contiguous organs to improve long-term survival in patients with LPS.^5,6,22,23^ In a single-institution study of 288 patients, Gronchi et al. found that an extended surgical resection was associated with a lower 5-year local recurrence rate and fewer metastases but with no significant improvement in OS or significant difference in morbidity. More so, there were limitations to this study. Patients were divided into two groups based on year of surgical intervention (before 2002 and after 2002) because of a systemic shift toward multivisceral resections that occurred in 2002.^5^ In another study of 382 patients from France, Bonvalet et al. found that systematic resection of uninvolved contiguous organs resulted in 3.29 times lower rate of recurrence; however, it was not associated with improvement in OS. Risk factors associated with decreased OS included high grade, tumor rupture, gross residual disease, and positive margins.^6^ MacNeill et al. described the combined experience of the Transatlantic Retroperitoneal Sarcoma Working Group (TARPSWG); in their analysis of 1007 patients, there was no significant association of extended surgical resection with surgical morbidity or mortality. However, age, need for blood transfusion, and weighted resected organ score were predictive of severe adverse events, which were defined as postoperative hematoma, anastomotic leak, abscess, sepsis, wound infection, bowel obstruction, fistula formation, venous thromboembolism, evisceration, respiratory failure, renal failure, or other. The weighted organ score was used to account for the anticipated morbidity associated with the complexity of the surgery rather than simply the number of organs resected; for example, resection of the appendix or gallbladder possessed a weight organ score of 0, whereas a pancreaticoduodenectomy possessed a weighted organ score of 2.^23^

Our study’s findings are more consistent with previous reports from North American centers. In a single institution, multivariate analysis of 83 patients, Ikoma et al. found that concomitant organ resection was not associated with a survival benefit in patients with RPS. There was no postoperative mortality in this study, defined as death within 30 days of surgical resection. Ultimately, the study advocated for the resection of contiguous organs only in the presence of direct invasion.^24^ In a multicenter, propensity score-matched analysis of 1139 patients, Villano et al. found no significant improvement in overall survival associated with radical resection (RR) after accounting for tumor grade, histology, hospital volume, and adjacent organ involvement. Like our study, RR was associated with increased morbidity without an associated improvement in OS.^14^

Despite advances in imaging modalities, the operating surgeon still combats the clinical challenge of resecting adjacent organs based on intraoperative concerns of invasion.^25,26^ Several studies have investigated the involvement of adjacent organs as determined by histologic invasion and the implications on recurrence and survival. In a study by Fairweather et al. 99 patients with resection of at least one additional organ for RPS were evaluated from 2002–2011. The rationale for clinical resection was taken into consideration and categorized based on suspected invasion or tumor origin, tumor adherence, resection required for R0/R1 resection, or other. The presence of histologic invasion was identified in 25% of the resected organs. Notably, histologic invasion was most observed in organs resected for the purpose of clinical suspicion of invasions rather than R0 resection. While subjectivity is a relevant factor that drives the selection for organ invasion, the histologic subtype of the RPS was a predictive factor of invasions, with the likelihood increased in dedifferentiated liposarcomas and leiomyosarcomas.^25^ In a retrospective study by Improta et al., 109 patients were evaluated for histologic organ involvement (HOI) after multivisceral resection of primary retroperitoneal liposarcomas between the years of 2009 and 2014. Approximately 40% of patients demonstrated infiltration of the capsular serosa or fascia or resected organs and viscera, and approximately 40% of patients experienced an infiltration of the malignant cells into the parenchyma or muscular layer of adjacent organs or structures. The study determined that the degree of HOI was a prognostic indicator for OS and DFS, supporting the benefit of multivisceral resection.^26^ Therefore, clinical suspicions of invasion are relevant points of consideration to account for during intraoperative resection.

While informative, the findings from our analysis should be interpreted within the context of the study's limitations. These include the retrospective nature of the study, which may introduce bias. Additionally, intraoperative tumor-related details including factors, such as degree of tumor invasion versus adherence versus abutment and intraoperative concern for invasion. Moreover, limitations of the NCDB include lack of justification for organ resection, presence and date of tumor recurrence, longitudinal treatment data, cause of mortality, and patient-reported adverse events. Importantly, the specific organ involvement and the number of adjacent organs resected could not be determined based on the NCDB dataset. Nonetheless, our analysis does provide effective data regarding our endpoints of 30-day and 90-day mortality, overall survival, and 30-day readmission after patients were matched based on age group, tumor grade, stage, adjacent tumor involvement, and Charlson-Deyo Comorbidity Index.

While it is widely regarded as a novel technique, gain in power is difficult to assess when using the matched win ratio approach, because there is not a clear consensus on how to perform a power analysis. In addition, there have been no software packages developed to perform this technique.^19^

## Conclusions

Multivisceral resection does not appear to provide an overall survival benefit in comparison to TO resection. Notably, a subgroup of patients, aged 72–90 years, who were analyzed in our study had a lower odds of winning or having a good outcome. Our results suggest that multivisceral resection increases morbidity without conferring an overall survival benefit. These findings highlight the need to further optimize patient selection and operative approach to improve patient outcomes. Ultimately, large-scale, multi-institutional prospective efforts must be undertaken to improve the understanding of the surgical management of this disease and to tailor treatment based on patient and tumor characteristics.

## Abbreviations

CC: Multivisceral resection
DM: Distant metastases
HOI: Histologic organ involvement
LPS: Liposarcoma
LR: Local recurrence
NCDB: National Cancer Database
OS: Overall survival
IQR: Interquartile range
WR: Win ratio
TARPSWG: Transatlantic Retroperitoneal Sarcoma Working Group
TO: Tumor-only resection
RR: Radical Resection
RPS: Retroperitoneal soft tissue sarcoma

## References

[1] Porter GA, Baxter NN, Pisters PWT (2006). Retroperitoneal sarcoma: a population-based analysis of epidemiology, surgery, and radiotherapy. Cancer..

[2] Wang J, Grignol VP, Gronchi A, Luo CH, Pollock RE, Tseng WW (2018). Surgical management of retroperitoneal sarcoma and opportunities for global collaboration. Chin Clin Oncol..

[3] Tseng WW, Seo HJ, Pollock RE, Gronchi A (2018). Historical perspectives and future directions in the surgical management of retroperitoneal sarcoma. J Surg Oncol..

[4] Matthyssens LE, Creytens D, Ceelen WP (2015). Retroperitoneal liposarcoma: current insights in diagnosis and treatment. Front Surg..

[5] Gronchi A, lo Vullo S, Fiore M, Mussi C, Stacchiotti S, Collini P (2009). Aggressive surgical policies in a retrospectively reviewed single-institution case series of retroperitoneal soft tissue sarcoma patients. J Clin Oncol..

[6] Bonvalot S, Rivoire M, Castaing M, Stoeckle E, le Cesne A, Blay JY (2009). Primary retroperitoneal sarcomas: a multivariate analysis of surgical factors associated with local control. J Clin Oncol..

[7] Oh SD, Oh SJ, Suh BJ, Shin JY, Oh CK, Park JK (2016). A giant retroperitoneal liposarcoma encasing the entire left kidney and adherent to adjacent structures: a case report. Case Rep Oncol..

[8] Singer S, Antonescu CR, Riedel E, Brennan MF (2003). Histologic subtype and margin of resection predict pattern of recurrence and survival for retroperitoneal liposarcoma. Ann Surg..

[9] Park JO, Qin L-X, Prete FP, Antonescu C, Brennan MF, Singer S (2009). Predicting outcome by growth rate of locally recurrent retroperitoneal liposarcoma: the one centimeter per month rule. Ann Surg..

[10] Chen J, Hang Y, Gao Q, Huang X (2021). Surgical diagnosis and treatment of primary retroperitoneal liposarcoma. Front Surg..

[11] Schmitz E, Nessim C (2022). Retroperitoneal sarcoma care in 2021. Cancers.

[12] Bonvalot S, Gronchi A, le Péchoux C, Swallow CJ, Strauss D, Meeus P (2020). Preoperative radiotherapy plus surgery versus surgery alone for patients with primary retroperitoneal sarcoma (EORTC-62092: STRASS): a multicentre, open-label, randomised, phase 3 trial. Lancet Oncol..

[13] Tseng WW, Barretta F, Conti L, Grignani G, Tolomeo F, Albertsmeier M (2021). Defining the role of neoadjuvant systemic therapy in high-risk retroperitoneal sarcoma: a multi-institutional study from the Transatlantic Australasian Retroperitoneal Sarcoma Working Group. Cancer..

[14] Villano AM, Zeymo A, Nigam A, Chan KS, Shara N, Unger KR (2020). Radical excision for retroperitoneal soft tissue sarcoma: a national propensity-matched outcomes analysis. Surgery.

[15] Bonvalot S, Raut CP, Pollock RE, Rutkowski P, Strauss DC, Hayes AJ (2012). Technical considerations in surgery for retroperitoneal sarcomas: position paper from E-Surge, a master class in sarcoma surgery, and EORTC-STBSG. Ann Surg Oncol..

[16] MacNeill AJ, Fiore M (2018). Surgical morbidity in retroperitoneal sarcoma resection. J Surg Oncol..

[17] Abdalla S, Montez-Rath ME, Parfrey PS, Chertow GM (2016). The win ratio approach to analyzing composite outcomes: an application to the EVOLVE trial. Contemp Clin Trials..

[18] Hyer JM, Diaz A, Pawlik TM (2022). The win ratio: a novel approach to define and analyze postoperative composite outcomes to reflect patient and clinician priorities. Surgery..

[19] Redfors B, Gregson J, Crowley A, McAndrew T, Ben-Yehuda O, Stone GW (2020). The win ratio approach for composite endpoints: Practical guidance based on previous experience. Eur Heart J..

[20] Hao Z, Parasramka S, Chen Q, Jacob A, Huang B, Mullett T (2023). Neoadjuvant versus adjuvant chemotherapy for resectable metastatic colon cancer in non-academic and academic programs. Oncologist..

[21] Vardell VA, Ermann DA, Tantravahi SK, Haaland B, McClune B, Godara A (2023). Impact of academic medical center access on outcomes in multiple myeloma. Am J Hematol..

[22] Pisters PWT (2009). Resection of some-but not all-clinically uninvolved adjacent viscera as part of surgery for retroperitoneal soft tissue sarcomas. J Clin Oncol..

[23] Macneill AJ, Gronchi A, Miceli R, Bonvalot S, Swallow CJ, Hohenberger P (2018). Postoperative morbidity after radical resection of primary retroperitoneal sarcoma. Ann Surg..

[24] Ikoma N, Roland CL, Torres KE, Chiang Y-J, Wang W-L, Somaiah N (2018). Concomitant organ resection does not improve outcomes in primary retroperitoneal well-differentiated liposarcoma: a retrospective cohort study at a major sarcoma center. J Surg Oncol..

[25] Fairweather M, Wang J, Jo VY, Baldini EH, Bertagnolli MM, Raut CP (2018). Surgical management of primary retroperitoneal sarcomas: rationale for selective organ resection. Ann Surg Oncol..

[26] Improta L, Pasquali S, Iadecola S, Barisella M, Fiore M, Radaelli S (2023). Organ infiltration and patient risk after multivisceral surgery for primary retroperitoneal liposarcomas. Ann Surg Oncol..

